# Cytopenias and Associated Factors in Patients Living With HIV on ARV Treatment in Cameroon: An Analytical Cross-Sectional Study

**DOI:** 10.1155/ah/3328539

**Published:** 2025-08-06

**Authors:** Josué Louokdom Simo, Romaric De Manfouo Tuono, Bendzigho Tatsiane Manewa, Maryline Njopwouo Seuko, Yolande Nathalie Matchein, Claude Tagny Tayou

**Affiliations:** ^1^Faculty of Health Sciences, University of Montagnes, Bangangté, West Cameroon, Cameroon; ^2^Faculty of Medicine and Biomedical Sciences, University of Yaoundé 1, Yaoundé, Cameroon; ^3^Bafoussam Regional Hospital Center, Bangangté, Cameroon

**Keywords:** anemia, associated factors, cytopenias, PLWHA, treatment

## Abstract

**Introduction:** Cytopenias are a frequent concern in the management of patients living with HIV/AIDS (PLWHA). The study objective was to determine the burden of anemia and cytopenia among PLWHA on antiretroviral treatment in the Cameroonian context and to identify the associated factors.

**Methods:** We conducted an analytical and cross-sectional study over 4 months. The study population consisted of PLWHAs on ARV treatment at the DREAM Center in Dschang. Blood samples were taken in EDTA and dry tubes. Complete blood counts, CD4 count, ferritin, and serum iron measurements were performed using flow cytometry, ELISA, and spectrophotometry methods. The results were recorded in an Excel spreadsheet and analyzed using SPSS statistical software.

**Results:** 198 PLWHAs on ARV treatment with extremes aged 15–75 years were included in this study, with a sex ratio of 0.48 in favor of women. The frequency of anemia was 32.32%. Triple therapy (3TC + TDF + DLV) was the most commonly used regimen (75.76%). The frequency of anemias was 32.32%, and they were mainly normocytic and normochromic. 8.08% of the population had leukopenia, and 9.09% had thrombocytopenia. Advanced immunodeficiency (CD4 level: 200–500 C/mm^3^) was identified as a predictive factor for anemia in PLWH in this study (OR = 2.88 [0.82–10.11]; *p* = 0.097).

**Conclusion:** Cytopenias in general, and anemias, in particular, are very common in patients with HIV/AIDS. These results raise the need for adequate follow-up of the sick population to limit the effects of these cytopenias for a healthier life of patients.

## 1. Introduction

According to the World Health Organization (WHO), anemia is the decrease in the ability of red blood cells to oxygenate tissues, resulting in a hemoglobin concentration that is lower than the body's physiological needs [1, 2]. Its definition mainly takes into account sex, age, and physiological state of the patient [3]. The WHO estimates that around 1.7 billion people worldwide are affected by anemia [4]. This situation affects the patient's health and can be life-threatening [5]. It is a real public health problem on a global scale because it is at the origin of a vicious obstacle to quality of life, mortality, morbidity, and scientific progress [6]. Anemia is one of the most common hematologic complications in people living with HIV/AIDS (PLWHA) [7–9].

Indeed, HIV-AIDS remains highly prevalent in Africa in general and in Cameroon in particular. In 2021, 27.9 million people were living with HIV, including 2.1 million deaths due to AIDS [10]. HIV prevalence among adults in Cameroon was 3.7%, including 5.0% among women and 2.3% among men, corresponding to approximately 500,000 adults living with HIV in Cameroon [11].

Several causes of anemia are reported during AIDS, including nutritional deficiency (deficiency of vitamin B12, folic acid, and iron), complications related to infections (bacterial, viral, and fungal), and the treatment that has a toxic effect on the cells of the bone marrow and others [12]. This is because the human immunodeficiency virus affects the immune system and hurts the body's hematopoietic system. For example, some authors have reported anemia, leukopenia, and thrombocytopenia during HIV infection [6, 13, 14]. Regarding the leukocyte lineage, the main reported hematological disorder is neutropenia for which authors have reported 5%–30% of neutropenia at the onset of infection and an elevation up to 57%–76% in the final stage of the disease [15–18]. Regarding the platelet lineage, the authors report about 4%–40% of cases of thrombocytopenia during HIV/AIDS [3].

However, the advent of triple antiretroviral therapy has led to an improvement in clinical terms in general and hematological parameters in particular in PLWHA on this treatment, as well as a decrease in the mortality rate [19]. In addition, depending on the type of antiretroviral therapy used, there could be a positive or even negative effect on hematological parameters. Indeed, many drugs used to treat the complications of the disease have myelosuppressive effects, such as zidovudine which is a common cause of cytopenia [20, 21]. For example, morphologically, normocytic normochromic anemia is the most frequent; however, studies also described high frequencies of macrocytosis (e.g. 55.1% and 32.2%), usually related to treatment with ZDV [6, 22]. ZDV can interfere with DNA replication and the cell division of erythroblasts, causing macrocytosis in mature erythrocytes. This phenomenon occurs because ZDV competes with natural deoxynucleoside triphosphates for binding to reverse transcriptase and deoxynucleoside triphosphates for incorporation into newly synthesized DNA viral strands, inhibiting viral reverse transcriptase and mammalian cellular DNA polymerase. The development of macrocytosis in PLWHA may signify adherence to ZDV antiretroviral regimens [6, 22–24].

All hematological conditions coupled with complications of the disease affect the patient's quality of life. To limit the harmful effects, antiretroviral treatment would improve the patient's quality of life and, indirectly, his hematological parameters. However, there are reports of cytopenias persisting during the disease despite treatment. Cytopenias affect treatment and accelerate disease progression [25]. Anemia and cytopenias remain a problem of concern particularly in countries with limited resources [9, 26]. The severity of cytopenias and anemias affects the life of the PLWH. Diagnosing anemias and cytopenias would allow for better therapeutic follow-up of patients. Although studies have been conducted on the study of cytopenias in AIDS, few studies in the Cameroonian context show the severity of hematological complications in HIV-positive patients, let alone those during antiretroviral treatment. Therefore, this study proposes to analyze the frequency of anemia and cytopenias in PLWHA on ARV treatment and to identify the factors associated with them to contribute to the improvement of patient care [27].

## 2. Methodology

### 2.1. Study Design and Study Population

We conducted a cross-sectional and analytical study over 04 months at the DREAM Center in Dschang and the Saint Vincent de Paul Hospital in West Cameroon. The DREAM Center in Dschang is one of the reference centers for the HIV care in Western Cameroon. We enrolled HIV-positive, treatment-naïve, and/or treatment-naïve patients receiving treatment. An estimate of the minimum sample size for this study was made from the arithmetic formula: *n*=*t*^2^ × *p* × (1 − *p*)/m^2^ [28], taking into account the prevalence of anemia in people (23%) previously reported by Melese et al. [6]. Using a preestablished form, we collected the clinical–biological and sociodemographic data of each selected participant including the date of diagnosis of the disease, the type of treatment, the duration of the infection, lifestyle habits, and comorbidities/co-infections. The patients unable to provide the required data and pregnant or lactating women, given the physiological changes they undergo during pregnancy and breastfeeding, were not included in order to avoid confounding factors. A total of 198 HIV-positive patients were included. Subsequently, the search for infection was confirmed by standard methods described further.

### 2.2. Laboratory Analysis of Patient's Samples

A blood volume of 04 mL was obtained from each participant in a dry tube and an EDTA tube for serological, immunological, and hematological tests, whose principles and methods are described as follows. Once the samples were collected, they were sent to the hematology, biochemistry, and immunoserology laboratories.

Before the immunoserology laboratory, confirmation of HIV infection was performed in each participant by enzyme-linked immunosorbent assay using the indirect ELISA principle (“Fortress Diagnostics,” United Kingdom). Subsequently, in hematology, a blood count was performed on each of the EDTA tubes (Mindray BC-5000, China) using a principle based on flow cytometry to perform the cell count, and the interpretation was performed according to the values defined by the WHO. Subsequently, the assessment of the shape and quality of the cells, and the reticulocyte count for the classification of anemia, if any, was made from a blood smear stained according to Romanowsky's principle [29]. The blood smear slides were read using an IRMERCO optical microscope. The determination of the CD4 rate was performed using the BD FACS machine based on flow cytometry, the principle of which is based on the use of an antibody labeled with fluorochrome, allowing the identification of these cells.

The evaluation of the patients' iron balance was based on the determination of ferritinemia by the enzyme-linked immunosorbent method using the indirect ELISA principle according to the protocol of the reagent “Elabscience^R^” of the batch number ER020N407677. Serum iron level was determined by the spectrophotometric method from DIALAB DTN-405 chemistry analyzer. Thus, serum iron and ferritin were interpreted for respective reference values of 0.5–1.2 g/L and 10–200 ng/mL according to the reference values defined by the different kits used (previously cited) and in correlation with the WHO values.

### 2.3. Data Management and Statistical Analysis

We defined and categorized anemia according to the WHO definition [30]. Thus, anemia has been defined in humans for Hb concentration < 13 g/dL; while in nonpregnant women, it was defined for a hemoglobin level < 12.0 g/dL [30]. From the definition of anemia, patients were categorized into “anemia patients” and “nonanemia patients.” Immune status was classified as severe immunodeficiency, advanced immunodeficiency, and no immunodeficiency for CD4 counts of < 200 cells/mm^3^, 200–499 cells/mm^3^, and ≥ 500 cells/mm^3^, respectively [31]. The data obtained were recorded using an Excel spreadsheet and analyzed by statistical software R Version 4.1.1. The qualitative variables studied were sociodemographic and clinicobiological characteristics such as patients' lifestyle and clinical characteristics, sex, occupation, marital status, comorbidities, treatment regimen, and interval over treatment duration; quantitative variables were age and hematological parameters including red blood cell count, white blood cell count, platelet, MCV, MCH, MCHC, CD4 count, serum iron, and ferritin. White blood cells, MCV, MCH, and MCHC were interpreted according to the following respective usual cutoff values: 4000–10,000/mL, 80–100 fL, 32–36 g/L, and 27–29 pg as recommended by WHO. Each of the quantitative variables was dichotomized into “low, normal, or high” according to the reference intervals defined by the reagent manufacturer or according to WHO.

Quantitative variables were presented as mean and qualitative variables as frequency. The comparison of quantitative variables was based on the Student's test and qualitative variables on the chi^2^'s test. Logistic regression analyses using univariate models were used to identify factors associated with infection in participants. The confidence level for this study was set at 5% for a value of *p*=0.05.

### 2.4. Ethical Considerations

For the conduct of this study, the ethical clearance (no. 2023/184/UdM/PR/CEAQ) and a research authorization (no. 2023/665/UdM/PR/DECANAT-ISSS/SMSB) have been obtained from the Ethics and Research Committee and the central administration of “Université des Montagnes.” In addition, authorization for collection and analysis has been obtained from the general administration of the DREAM Center of Dschang and the Saint Vincent de Paul Hospital, West Cameroon. This study was carried out in strict compliance with the principles of research according to the Declaration of Helsinki. The study population was previously informed about the benefits and risks of the research, the progress of the research, and the expected objectives through a technical sheet that was given to them. Eligible patients gave their free and informed consent by signature. The data obtained from the participants were kept confidential [32].

## 3. Results

198 PLWHAs were included in this study. 64 patients (32.32%) were anemic. The mean age of the study population was 41.54 ± 13.37 years, of which 43.35 ± 13.86 years were anemic and 38.03 ± 11.87 years were nonanemic.

### 3.1. Distribution of the Study Population by Sociodemographic and Clinicobiological Characteristics

#### 3.1.1. Sociodemographic Characteristics

Table 1 describes the sociodemographic characteristics of the study population.

It can be seen from Table 1 that 64 patients (32.32%) were male compared to 134 patients (67.68%), who were female with a sex ratio (M/F) of 0.48. Also, there was no significant association between these demographic social parameters and anemia.

#### 3.1.2. Clinicobiological Characteristics

Table 2 describes the clinicobiological characteristics of the study population.

From Table 2, it can be seen that 48.48% of the population had been on treatment over an average period of 0–10 years. 75.76% of the population used the first-line lamivudine/tenofovir/dolutegravir (3TC/TDF/DLV) regimen. There is a significant association between the nature of the protocol and the occurrence of anemia.

### 3.2. Population Distribution Based on Biological Data

#### 3.2.1. Hematological Data

##### 3.2.1.1. Red Lineage, Anemia, and Biological Characterization

Table 3 describes the profile and frequency of red-line blood count disorders, anemia, and biological characterization in the population.

Table 3 shows that 32.32% of the population had a low hemoglobin level compared to the mean hemoglobin of 10.63 g/dL ± 1.20 in anemic patients and 13.41 g/dL ± 1.22 in nonanemic patients (*p* < 0.0001). The mean red blood cell count was 3.90 T/L ± 0.51 in anemic patients and 4.44 T/L ± 0.45 in nonanemic patients (*p* < 0.0001).

##### 3.2.1.2. White and Platelet Lineage

Table 4 describes the profile and frequency of white and platelet line hemogram disorders in the population.

From Table 4, the mean platelet count was 214.33 G/L ± 71.95, of which 9.09% of the population had low platelet counts. In addition, the mean white blood cell count was 5277.78 ± 1578.98/μL, with 8.08% of patients having a low white blood cell count.

#### 3.2.2. Biochemical Data and Immune Status

Table 5 describes the biochemical and immunological characteristics of the study population.

Table 5 above shows that the mean serum iron was 69.1 ± 38.1 μg/dL in anemic patients and 89.2 ± 23.0 μg/dL in nonanemic patients (*p*=0.001). Moreover, the mean serum ferritin was 213 ± 31.5 ng/mL in anemic patients and 275 ± 19.7 ng/mL in nonanemic patients (*p*=0.069). 34.4% of anemic patients had a CD4 count between 200 and 500 C/mm^3^ (advanced immunodeficiency) compared to 22.4% in nonanemic patients (*p*=0.019).

## 4. Factors Associated With Anemia

Table 6 describes the factors associated with anemias in the population during the study.

From Table 6, advanced immunodeficiency (CD4 count 200–500 C/mm^3^) was identified as a predictive factor of anemia in PLWH during this study (OR = 2.88 [0.82–10.11]; *p*=0.097).

## 5. Discussion

This study aimed to report on the frequency of anemia and cytopenias in PLWHA on ARV treatment and to identify the factors associated with the latter. Indeed, a variety of hematologic abnormalities associated with HIV have been reported in several studies. They report that these hematological abnormalities constitute a real public health problem in the world in general and in Sub-Saharan Africa in particular, including several countries, including Cameroon. They report that anemias are the most common [33]. Thus, this study reports a high frequency of cytopenias in AIDS, mainly characterized by anemia, of which 32.32% were reported. The results of our study show a high prevalence of anemia among PLWH in Cameroon, confirming data in the literature. This anemia is predominantly normocytic normochromic, suggesting a multifactorial mechanism involving both central (spinal cord injury) and peripheral (hemorrhage and hemolysis) causes [16, 24]. Iron deficiency, observed in a significant proportion of our patients, could play an important role in the pathogenesis of this anemia [34]. In addition, chronic inflammation associated with HIV infection may impair erythropoiesis and contribute to anemia [35, 36]. These findings underline the importance of regular hematological monitoring and appropriate management of nutritional deficiencies in PLWH. These results are close to the 34% reported in northern Ethiopia (35%) [33] and Central Ethiopia (33%) [37], in Rwanda (29%) [38]. These results are lower than those reported in Tanzania (77.4%) [39] and China (51.9%) [40]. However, they are higher than those reported in Nigeria (24.3%) [41], India (16.2%) [42], and Ghana (23.8%) [43]. The variation in results with previous studies may be explained by disparities in sociodemographic and immune characteristics but is still evidence of a significant frequency of anemia during HIV/AIDS. Anemias were mainly normocytic (82.83%), normochromic (81.82%), and regenerative (15.15%) and were also reported by other authors [2, 5]. Furthermore, the reported anemias were mainly regenerative. The authors also report mainly hyporenegenerative anemias in PLWHA. Indeed, hemolysis may also be responsible for anemia development in PLWHA. The formation of anti-erythrocyte antibodies as a consequence of hypergammaglobulinemia is the main cause of hemolytic anemia in PLWHA. Other causes of hemolytic anemia in PLWHA are microangiopathic hemolytic anemia and drug-induced anemia in patients who present other comorbidities [24, 34, 44].

In this study, 64 patients (32.32%) were male versus 134 female patients (67.68%) with a sex ratio (M/F) of 0.48. This predominance of women can be explained, not only by the presence of a genital contact surface that is more exposed to recurrent infections, making women vulnerable to this infection, but also by the greater use of these infections in health facilities [45]. This female predominance has been reported by several authors [26, 33, 46]. This observation is in line with the results of other studies conducted in the region [26, 33, 46]. This overrepresentation of women in the HIV-infected population is multifactorial. On the one hand, anatomical and behavioral factors put women at greater risk of infection. On the other hand, sociocultural and economic inequalities, which limit women's access to care and sex education, also play a decisive role.

The average age of our population was 41.54 ± 13.27 years with extremes ranging from 15 to 75 years. The most represented age group was between 30 and 45 years old; an age group that is primarily the age group in which young people are sexually active and therefore vulnerable to HIV [47]. Several authors also reported a similar average age and age range during their studies [33, 46]. The mean age of our population, which is 41.54 years, with a majority of patients aged between 30 and 45, is in line with HIV epidemiological data, which show a peak prevalence in this age group, particularly linked to risky sexual behavior. These results underline the need to reinforce prevention interventions targeting young adults, with an emphasis on sex education and the promotion of voluntary HIV testing.

The majority use of the 3TC + TDF + DLV regimen in our study reflects current WHO recommendations for first-line antiretroviral therapy [48]. By targeting different stages of the viral replication cycle, this triple combination optimizes therapeutic efficacy and delays the emergence of resistance [49]. The results observed are comparable to those reported by Waters et al. [50], but lower than those of Karakodjo et al. [45], which could be explained by differences in epidemiological contexts and current therapeutic policies. The fact that almost half of the population studied had been on treatment for less than 10 years suggests a recent epidemic dynamic in the region. This observation underscores the need to pursue HIV prevention and early detection efforts.

Regarding the immune status of patients, the CD4 count was mostly < 200 C/mm^3^ in the population (65.67%), reflecting severe immunodeficiency. Several authors also report a large proportion of patients with CD4 counts < 200 C/mm^3^ during their study [7, 51, 52]. Indeed, the CD4 count is one of the important elements in human physiology that would have important biological implications during the disease because it is notably associated with the worsening of the pathophysiology. For example, patients with low CD4 counts (< 200 C/mm^3^ and/or 200–500 mm^3^) had a higher susceptibility to develop anemia compared to those with CD4 counts > 500 mm^3^ (*p* = 0.019). These findings have also been made by authors of studies conducted in Ethiopia [49, 53]. Indeed, the deterioration of hemoglobin formation in patients is the result of disrupted hematopoiesis due to high production of inflammatory cytokines and a decrease in the production of hematopoietic growth factors associated with poor absorption and alteration of the recycling of the ferruginous substance secondary to HIV-AIDS [54]. Moreover, the decrease in CD4 counts is correlated with the decrease in the immunity of patients exposing them to opportunistic infections, which are important factors in the distribution of micronutrients, which in this case is iron [55]. Thus, this study reports that 18.75% of anemic patients had a decrease in serum iron compared to none in those without anemia (*p* < 0.001); furthermore, the mean ferritin in anemic and nonanemic patients during this study was 213 ± 31.5 and 275 ± 19.7 ng/mL, respectively (*p* = 0.069). Obirikorang et al. also reported a decrease in serum iron and ferritin in anemic AIDS patients compared to those without anemia during their study (*p* = 0.0019) and (*p* = 0.0021), respectively [43]. The respective decreases in serum iron and ferritin in anemic versus nonanemic patients would suggest a iron deficiency in the latter, although further results in further studies are needed to confirm this claim.

Other cytopenias reported during this study were leukopenia and thrombocytopenia. Leukopenia was observed in 8.08% of the population, including 10.45% in nonanemia and 3.13% in anemia (*p*=0.19). Leukopenia is characterized by lymphopenia in the study population (9.09%), including 15.63% in anemia patients and 5.97% in nonanemia patients. Moreover, this study reports 9.09% of monocytopenias, including 15.63% in anemic PLWHA and 5.97% in those without anemia. Authors such as Bhardwaj et al. reported 18.33% leukopenia, 49.17% lymphopenia, and 5% neutropenia in PLWHA [56]. Several other authors report white lineage cytopenias in PLWHA [17, 18, 56]. In addition, 9.09% of thrombocytopenias were reported during this study. Bhardwaj et al. reported 15.83% thrombocytopenia in PLWHA [56]. Melese et al. in a meta-analysis also reported frequent thrombocytopenia in PLWHA, as well as several other authors [6, 42, 57]. As reported above, thrombocytopenia and leukopenia are the result of bone marrow–induced destruction by viral activity and immunological factors [58]. The presence of cytopenias, notably leukopenia and thrombocytopenia, in our patients underscores the hematological complexity of HIV infection. These cellular abnormalities are frequently reported in the literature [6, 17, 18, 42, 56, 57]. Leukopenia, characterized by lymphopenia in our study, is probably related to virus-induced lymphocyte destruction and severe immunosuppression. Thrombocytopenia, on the other hand, could result from viral platelet destruction or bone marrow dysfunction.

Advanced immunodeficiency (CD4 level: 200–500 C/mm^3^) was identified as a predictive factor for anemia in PLWH in this study (OR = 2.88 [0.82–10.11]; *p*=0.097). The significant association between anemia and low CD4 count confirms the findings of other studies [7, 8, 54]. Profound immunodepression contributes to the onset of anemia through various mechanisms, including dysregulation of erythropoiesis and increased inflammation.

A key limitation of this cross-sectional study is its inability to establish causality between antiretroviral treatment and hematological disorders. While this study provides valuable insights into the prevalence and associated factors of cytopenias among PLWHA, a longitudinal cohort study is warranted to investigate the temporal relationship between treatment initiation and the development of these complications; among other things, it would also allow us to have information on the second line of ARV treatment taken by patients. Such a study would allow for a more comprehensive understanding of the long-term impact of antiretroviral therapy on hematological health. Another limitation of this study is that although the gender distribution was representative, it may limit generalizability to settings with different HIV demographics.

## 6. Conclusion

This study underscores the high prevalence of cytopenias, particularly anemia, among individuals living with HIV/AIDS in our study population. Our findings highlight the critical role of CD4 count, serum iron, and ferritinemia in the development of anemia. While the association between antiretroviral therapy and anemia was not evident in this study, public health authorities and healthcare providers need to prioritize routine screening, early diagnosis, and appropriate management of cytopenias in PLWH. By implementing comprehensive care plans, including nutritional interventions and regular monitoring, we can significantly improve the quality of life for these patients.

## Figures and Tables

**Table 1:** Sociodemographic characteristics of the study population.

Parameters		Status			
		NAP *N* = 134 *n* (%)	AP *N* = 64 *n* (%)	Total = 198 *n* (%)	*p* value
Sex	Male	52 (38.81)	12 (18.75)	64 (32.32)	0.008
	Female	82 (61.19)	52 (81.25)	134 (67.88)	

Age *M* ± Sd		43.35 ± 13.86	38.03 ± 11.87	41.54 ± 13.37	0.007

Marital status	Single	24 (17.91)	14 (21.9)	38 (19.19)	0.29
	Divorced	8 (5.97)	6 (9.38)	14 (7.07)	
	Married	64 (47.76)	28 (43.75)	92 (46.46)	
	Common-law union	20 (14.93)	12 (18.75)	32 (16.16)	
	Widower	18 (13.43)	4 (6.25)	22 (11.11)	

*Note: n*, effective.

Abbreviations: AP, anemia patients; *M*, mean; NAP, nonanemic patients; Sd, standard deviation.

**Table 2:** Clinicobiological characteristics of the study population.

Parameters		Status			
		NAP *N* = 134 *n* (%)	AP *N* = 64 *n* (%)	Total = 198 *n* (%)	*p* value
Duration of treatment (in a year)	[0–10]	64 (47.76)	32 (50.00)	96 (48.48)	0.48
	[11–20]	64 (47.76)	28 (43.75)	92 (46.46)	
	[21–30]	6 (4.48)	4 (6.25)	10 (5.05)	

Comorbidities	Hepatitis B	10 (7.46)	8 (12.5)	18 (9.1)	0.47
	Myoma	2 (3.13)	4 (6.25)	6 (3.03)	
	Malaria	0 (0.00)	2 (3.13)	2 (0.01)	
	None	124 (92.54)	56 (87.5)	180 (90.9)	

Regimen	3TC/TDF/DLV	100 (74.63)	50 (78.13)	150 (75.76)	0.06
	3TC/TDF+AT/RT	18 (13.43)	6 (9.38)	24 (12.12)	
	3TC/TDF/EFV	14 (10.45)	8 (12.50)	22 (11.11)	
	ABC/3TC/DLV	2 (1.49)	0 (0.00)	2 (1.01)	

Other treatment	Insulin	2 (1.49)	2 (3.13)	4 (2.02)	0.33
	Ketoconazole	0 (0.00)	2 (3.13)	2 (1.01)	
	Native treatment	0 (0.00)	2 (3.13)	2 (1.01)	
	None	132 (97.54)	58 (86.40)	190 (95.96)	

*Note: n*, effective; 3TC, lamivudine; TDF, tenofovir; DLV, dolutégravir; AT, atazanavir; RT, ritonavir; EFV, éfavirenz; ABC, abacavir; comorbidities = hepatitis B, myoma, malaria.

Abbreviations: AP, anemia patients; NAP, nonanemic patients.

**Table 3:** Profile and frequency of red lineage disorders, anemia, and biological characterization.

Parameters		Status			
		NAP *N* = 134 *n* (%)	AN *N* = 64 *n* (%)	Total 198 *n* (%)	*p* value
Hemoglobin *M* ± Sd		13.41 ± 1.22	10.63 ± 1.20	12.50 ± 1.78	< 0.0001⁣^∗^
Hemoglobin	Normal	134 (100.00)	0 (0.00)	134 (67.68)	0.71
	Low	0 (0.00)	64 (100.00)	64 (32.32)	

RBC *M* ± Sd		4.44 ± 0.45	3.90 ± 0.51	4.26 ± 0.53	< 0.0001⁣^∗^
RBC	Normal	118 (88.06)	40 (62.50)	158 (79.80)	0.28
	Low	16 (11.94)	24 (37.50)	40 (20.20)	

Hematocrit (*M* ± Sd)		40.09 ± 3.80	32.87 ± 4.10	37.75 ± 5.15	< 0.0001⁣^∗^
Hematocrit	Normal	128 (95.52)	20 (31.25)	148 (74.75)	0.57
	Low	6 (4.48)	44 (68.75)	50 (25.25)	

MCV (*M* ± Sd)		91.64 ± 6.32	86.52 ± 6.41	89.97 ± 6.76	0.0002⁣^∗^
MCV	High	24 (17.91)	4 (6.25)	28 (14.14)	0.19
	Normal	108 (80.60)	56 (87.50)	164 (82.83)	
	Low	2 (1.49)	4 (6.25)	6 (3.03)	

MCHC (*M* ± Sd)		33.22 ± 1.53	31.82 ± 3.01	32.79 ± 2.21	0.002⁣^∗^
MCHC	Normal	128 (94.04)	38 (56.25)	166 (81.83)	0.42
	Low	6 (4.48)	24 (37.50)	30 (15.15)	

MCH (pg)		30.48 ± 2.49	27.59 ± 3.80	29.55 ± 3.26	< 0.0001⁣^∗^
MCH	Normal	134 (100)	54 (81.25)	188 (87.88)	0.16
	Low	0 (0.00)	10 (15.63)	10 (5.05)	

Reticulocytes (*M* ± Sd)		87.55 ± 31	45 ± 12	66.3 ± 215	0.23
Reticulocytes	Normal	/	58 (90.6)	58 (29.30)	0.70
	Low	/	6 (4.48)	6 (3.03)	

*Note:* Hemoglobin (g/dL); RBC (T/L); hematocrit (%); MCH,  mean corpuscular hemoglobin content (pg); reticulocytes (G/L); no stratification by sex because the definitions of microcytosis and hypochromia are not specific to a particular sex.

Abbreviations: AP, anemia patients; *M*, mean; MCHC, mean corpuscular hemoglobin concentration (g/dL); MCV, mean corpuscular volume (fL); NAP, nonanemic patients; Sd, standard deviation.

^∗^Significant difference at *p* < 0.05.

**Table 4:** Profile and frequency of white and platelet lineage disorders.

Parameters		Status			
		NAP *N* = 134 *n* (%)	AP *N* = 64 *n* (%)	Total 198 *n* (%)	*p* value
WBC (*M* ± Sd)		5150.75 ± 1117.40	5543.75 ± 2261.73	5277.78 ± 1578.98	0.25
WBC	High	0 (0.00)	2 (3.17)	2 (1.01)	0.19
	Normal	120 (89.55)	60 (93.75)	180 (90.91)	
	Low	14 (10.45)	2 (3.13)	16 (8.08)	

Lymphocytes (*M* ± Sd)		2586.57 ± 926.04	2378.13 ± 1188.04	2519.19 ± 1016.66	0.34
Lymphocytes	High	10 (7.46)	6 (9.38)	16 (8.08)	0.16
	Normal	116 (86.57)	48 (75.00)	164 (82.83)	
	Low	8 (5.97)	10 (15.63)	18 (9.09)	

Monocytes (*M* ± Sd)		317.91 ± 182.51	321.88 ± 259.14	319.19 ± 209.31	0.93
Monocytes	High	2 (1.49)	2 (3.13)	4 (2.02)	0.17
	Normal	124 (92.54)	52 (81.25)	176 (88.9)	
	Low	8 (5.97)	10 (15.63)	18 (9.09)	

Granulocytes (*M* ± Sd)		2255.22 ± 870.26	2825.00 ± 1645.13	2439.39 ± 1199.13	0.02^∗^
Granulocytes	High	0 (0.00)	2 (3.13)	2 (1.01)	0.21
	Normal	76 (56.72)	46 (71.88)	122 (61.62)	
	Low	58 (43.28)	16 (25.00)	74 (37.37)	

Platelet (*M* ± Sd)		200.656 ± 52.86	242.875 ± 95.78	214.33 ± 71.95	0.005⁣^∗^
Platelet	High	0 (0.00)	2 (3.13)	2 (1.01)	0.20
	Normal	118 (88.06)	60 (93.75)	178 (89.90)	
	Low	16 (11.94)	2 (3.13)	18 (9.09)	

*Note:* Lymphocytes (/μL); monocytes (/μL); granulocytes (/μL); platelets (G/L); reticulocytes (G/L); no stratification of the blood count profile by sex because the definition of associated disorders is not specific to a particular sex.

Abbreviations: AP, anemia patients; NAP, nonanemic patients; WBC, white blood cell (/μL).

^∗^Significant difference at *p* < 0.05.

**Table 5:** Biochemical data and immune status of the study population.

Parameters		Status			
		NAP *N* = 134 *n* (%)	AP *N* = 64 *n* (%)	Total = 198 *n* (%)	*p* value
Creatinine (*M* ± Sd)		0.99 ± 0.17	0.94 ± 0.14	0.97 ± 0.16	0.044
Créatinine	High	2 (1.49)	0 (0.00)	2 (1.01)	0.23
	Normal	120 (89.55)	62 (96.88)	182 (91.92)	
	Low	12 (8.96)	2 (3.13)	14 (7.07)	

Serum iron (*M* ± Sd)		89.2 ± 23.0	69.1 ± 38.1	79.15 ± 30.55	< 0.001^∗^
Serum iron	High	0 (0.00)	2 (3.13)	2 (1.01)	0.37
	Normal	134 (100.00)	50 (78.13)	184 (92.93)	
	Low	0 (0.00)	12 (18.75)	12 (6.06)	

Ferritin (*M* ± Sd)		275 ± 19.7	213 ± 31.5	244 ± 25.6	< 0.001⁣^∗^
CD4	< 200	92 (68.7)	38 (59.4)	130 (65.67)	0.019
	200–500	30 (22.4)	22 (34.4)	52 (26.26)	
	> 500	12 (8.96)	4 (6.25)	16 (8.07)	

*Note:* Creatinine (mg/dL); serum iron (μg/dL); ferritin (ng/mL); CD4 (C/mm^3^); reticulocytes (G/L).

Abbreviations: AP, anemia patients; NAP, nonanemic patients.

^∗^Significant difference at *p* < 0.05.

**Table 6:** Factors associated with anemias in the population.

Parameters		Nonanemic *N* = 134 *n* (%)	Anemia *N* = 64 *n* (%)	Univariate analysis OR [CI 95%]	*p* value
Sex	Male	52 (38.81)	12 (18.75)	Ref.	Ref.
	Female	82 (61.19)	52 (81.25)	0.44 [0.21; 0.91]	0.024

Duration of treatment	[0–10]	64 (47.76)	32 (50.00)	Ref.	Ref.
	[11–20]	64 (47.76)	28 (43.75)	0.96 [0.51; 1.80]	0.893
	[21–30]	6 (4.48)	4 (6.25)	1.62 [0.42; 6.18]	0.494

Regimen	3TC/TDF/DLV	100 (74.63)	50 (78.13)	Ref.	Ref.
	3TC/TDF+AT/RT	18 (13.43)	6 (9.38)	0.75 [0.28; 2.02]	0.596
	3TC/TDF/EFV	14 (10.45)	8 (12.50)	0.85 [0.31; 2.31]	0.770
	ABC/3TC/DLV	2 (1.49)	0 (0.00)	0.00 [0.00;.]	0.485

Creatinine (*M* ± Sd)		0.99 ± 0.17	0.94 ± 0.14	0.13 [0.02; 0.90]	0.038
Low		12 (8.96)	2 (3.12)	Ref.	Ref.
High		2 (1.49)	0 (0.00)	0.00 [0.00]	0.758
Normal		120 (89.6)	62 (96.9)	3.10 [0.67; 14.3]	0.133

Serum iron (μg/dL), (*M* ± Sd)		89.2 ± 23.0	69.1 ± 38.1	0.98 [0.97; 0.99]	< 0.001
Low		0 (0.00)	12 (18.8)	Ref.	Ref.
High		0 (0.00)	2 (3.12)	. [.;.]	1.000
Normal		134 (100)	50 (78.1)	0.00 [0.00]	< 0.001

Ferritin, (M ± Sd)		275 ± 19.7	213 ± 31.5	0.93 [0.91; 0.95]	< 0.001
CD4	< 200	92 (68.7)	38 (59.4)	0.81 [0.24; 2.66]	0.756
	200–500	30 (22.4)	22 (34.4)	2.88 [0.82–10.11]	0.097
	> 500	12 (8.96)	4 (6.25)	Ref.	Ref.

WBC (*M* ± Sd)		5150.75 ± 1117.4	5543.75 ± 2261.7	1.00 [1.00; 1.00]	0.336

Platelets (*M* ± Sd)		200.65 ± 52.86	242.875 ± 95.78	1.00 [1.00; 1.00]	0.002

*Note: n*, effective; Ref: 1.

Abbreviations: CI, confidence interval; OR, odds ratio.

^∗^ *p* < 0.05.

## Data Availability

The data that support the findings of this study are available from the corresponding author upon reasonable request.
